# Access and Attrition Across Audiology and Cochlear Implant Care Among Older Adults with Bilateral Sensorineural Hearing Loss

**DOI:** 10.1097/ONO.0000000000000096

**Published:** 2026-08-11

**Authors:** Payal V. Patel, Varun Gopal, Divya Patel, Ryan G. Porter

**Affiliations:** 1 Carle Illinois College of Medicine, University of Illinois at Urbana-Champaign, Urbana, Illinois; 2 Department of Otolaryngology–Head and Neck Surgery, Carle Foundation Hospital, Urbana, Illinois.

**Keywords:** Audiology, Cochlear implant, Sensorineural hearing loss, Social determinants of health

## Abstract

**Objective:**

To quantify progression through the hearing care pathway among US adults aged ≥65 years with bilateral sensorineural hearing loss (SNHL) and describe variation across geographic and community contexts.

**Study Design:**

Cross-sectional study.

**Setting:**

Epic Cosmos Database, January 1, 2021, through December 31, 2024.

**Patients:**

Adults aged ≥65 years with bilateral SNHL identified using International Classification of Diseases, Tenth Revision codes.

**Main Outcome(s) and Measure(s):**

Stage-specific unadjusted odds ratios with 95% confidence intervals were calculated for progression across audiology evaluation, cochlear implant evaluation, and cochlear implantation. Statistical significance was defined as *P* < 0.05, with *P* < 0.001 considered a strong signal given the large sample size.

**Results:**

Among 1,461,489 older adults with bilateral SNHL, 746,118 (51.1%) underwent audiology evaluation; fewer than 2% received cochlear implant candidacy evaluation, while approximately 64% of those evaluated for cochlear implantation proceeded to surgery. Marked stage-specific attrition was observed. Adults aged 85 years or older had lower odds of audiology access, higher odds of candidacy evaluation, and lower odds of cochlear implantation. Black and Asian patients had lower odds of candidacy evaluation. Women were less likely to undergo candidacy evaluation but more likely to receive implantation once evaluated for candidacy. Patients living farther from a cochlear implant center and those in rural areas had higher odds of both candidacy evaluation and cochlear implantation, a counterintuitive finding that likely reflects a selection effect among patients who overcome distance barriers rather than improved geographic access.

**Conclusions:**

In this national cohort, disparities along the cochlear implant care pathway were associated less with audiologic access due to distance and more with attrition at cochlear implant candidacy assessment. Candidacy referral and evaluation represent a critical, potentially modifiable bottleneck for improving equity in hearing care among older adults.

Hearing loss is one of the leading causes of disability, affecting approximately 1.57 billion people worldwide and an estimated 79.9 million people in the United States as of 2021 (1). Prevalence increases sharply with age; nearly 1 in 3 US adults aged 65–74 and almost half of adults aged ≥75 years experience hearing loss (2,3).

Age-related hearing loss, or progressive bilateral sensorineural hearing loss (SNHL), impacts far more than communication alone. Untreated hearing loss has been associated with increased risk of dementia, depression, falls, social isolation, and cardiovascular disease (4–9).

Cochlear implants (CIs) are the standard of care for adults with significant SNHL who receive limited benefit from hearing aids and have been shown to improve communication ability, quality of life, and long-term health outcomes while being cost-effective (10–13). CI utilization in the United States remains low, with estimates ranging from 5% to 13% of eligible adults, and disparities documented across sociodemographic and regional contexts (14–16).

Adults with SNHL typically enter the hearing care pathway through diagnostic audiology evaluation, where hearing thresholds and functional communication are assessed. American Cochlear Implant Alliance recommendations emphasize CI evaluation referral when the unaided pure-tone average is ≥60 dB, and the word recognition score is ≤60% in the better ear (17,18). Once eligibility is met, patients progress through a defined care pathway from diagnostic audiology evaluation, advancing to candidacy assessment and surgery. However, recent national claims analyses report that cochlear implantation does not consistently align with diagnostic audiology utilization (14). Cochlear implantation had a weak correlation with SNHL and audiology utilization rates in that analysis, and this misalignment could imply that under-referral and limited clinical awareness, rather than absent diagnostic access, contribute to CI underutilization even in regions where hearing evaluations are common. This gap at the transition from diagnostic audiology to candidacy referral is the specific point the present study seeks to contextualize.

Among patients who do receive a CI candidacy evaluation, progression to surgery is also uneven. Prior work has shown that older patients, those with lower socioeconomic status, and those living outside metropolitan areas are less likely to undergo cochlear implantation, even when candidacy is confirmed (19,20). This indicates that a critical bottleneck exists not solely at the point of clinical eligibility determination, but also within the transition from CI evaluation to the decision-to-surgery stage of the care pathway. However, existing studies have largely examined cochlear implantation at isolated points of care. To our knowledge, national analyses have not characterized where along the CI pathway care disparities emerge, limiting the ability to distinguish barriers to audiology entry, candidacy evaluation, and implantation.

This study investigates the CI care pathway from audiology evaluation through candidacy assessment and implantation on a national scale. By examining associations between sociodemographic and geographic factors in a stage-specific progression, the analysis aims to identify potential structural bottlenecks that limit equitable access to cochlear implantation.

## MATERIALS AND METHODS

### Data Source

This cross-sectional study utilized Epic Cosmos (21), a Health Insurance Portability and Accountability Act—defined limited dataset that aggregates electronic health records and includes over 300 million patients from 1884 hospitals and 42,400 clinics across the United States, Canada, Lebanon, and Saudi Arabia (22). The database includes patient demographics, diagnoses, procedures, and socioeconomic factors. In accordance with institutional policy and federal regulations (45 CFR §46.102), this research was determined to be exempt from institutional review board approval. This study follows the STROBE reporting guidelines for cross-sectional studies (23).

### Study Population

A base patient was defined as a US resident aged 65 years or older who had at least 2 encounters in any 2-year timespan between January 1, 2021, and December 31, 2024, to ensure analysis of longitudinal patient charts. Within this base population, individuals with bilateral SNHL were identified using International Classification of Diseases, Tenth Revision (ICD-10) codes H91.13 (Presbycusis, bilateral) and H90.3 (SNHL, bilateral). Consistent with established CI candidacy criteria for this age group and to focus the analysis on age-related hearing loss for clarity of data, patients with conductive or mixed hearing loss, sudden or unilateral SNHL, and otologic conditions amenable to medical or non-CI surgical correction were excluded (16,24–26). These etiologies involve diagnostic work-up and management that differ from age-related bilateral SNHL and typically follow distinct, often non-CI, treatment pathways, which could confound a utilization analysis focused on the age-related hearing loss population for whom cochlear implantation is the relevant intervention. These exclusion criteria favor etiologic homogeneity over sensitivity. Detailed exclusion codes are provided in Supplemental Table 1, https://links.lww.com/ONO/A47.

CI status was defined using medical billing codes such as Current Procedural Terminology (CPT) codes and ICD-10 codes for an active CI device as outlined in Supplemental Table 2, https://links.lww.com/ONO/A48 (27–30).

Audiology evaluation was identified using CPT codes for diagnostic audiological examination or comprehensive threshold and speech recognition testing that capture completed billable services within the Epic Cosmos database (Supplemental Table 3, https://links.lww.com/ONO/A49) (14,31,32). These evaluations represent the clinical step typically preceding CI candidacy evaluation and serve as a measure of access to specialty diagnostic care rather than a measure of hearing severity.

CI candidacy evaluation, CPT code 92626 (15), captures entry into the CI evaluation pathway and formal assessment for potential implantation. As audiometric thresholds are not available in Epic Cosmos, this code represents access to evaluation rather than determination of CI eligibility.

### Covariates

Care pathway progression was analyzed across each stage: audiology evaluation, CI candidacy evaluation, and cochlear implantation. The patient groups were stratified by age at visit, sex recorded using legal documentation, self-reported race and ethnicity, US Census region, insurance, rural–urban commuting area (RUCA), and social vulnerability index (SVI).

RUCA codes are based on population density and urbanization patterns at the ZIP code level. RUCA codes were categorized into a 3-tier classification: metropolitan (primary RUCA code, 1–3), micropolitan (4–6), and rural communities (7–10) (33–35).

SVI was assigned according to each patient’s most recent residential ZIP code, using Census tract-level data from the US Centers for Disease Control and Prevention. SVI reflects community-level social and structural conditions, including income, transportation access, and housing characteristics (36–38). SVI was categorized into tertiles: 0 to <33% (lowest vulnerability), ≥33 to <66%, and ≥66% (highest vulnerability).

### Proximity to Cochlear Implant Surgical Centers

As the distance to the surgical center could impact the ability and decision to receive a CI (39), adult CI surgical centers were identified using publicly available center lists from *Advanced Bionics* and *Cochlear Americas* as of October 2025. This list aims to capture most US implanting institutions but may miss centers that are exclusively affiliated with other manufacturers such as MED-EL, though they are unlikely to significantly affect geographic classification.

A manual review of each location was conducted to confirm that it provided adult cochlear implantation. Locations offering audiology or programming services only, centers without a CI surgeon, and clinics serving exclusively pediatric populations were excluded. Veterans Affairs centers that provide cochlear implantation were included. The final list of CI surgical centers included is provided in Supplemental Table 4, https://links.lww.com/ONO/A50.

Geographic proximity to CI centers was represented using centroid-based geospatial distance calculations at a ZIP code level, using the geocode Python package. Distance was used as a measure of structural geographic access, not appointment availability or referral practices. Patients were categorized into 3 proximity strata: ≤25 miles (local), >25 to ≤50 miles (regional), and >50 miles (extended).

### Statistical Analysis

For stage-specific analyses, odds ratios (OR) with 95% confidence intervals were calculated using stage-specific denominators that reflect progression through the CI care pathway. For CI candidacy evaluation, analyses were restricted to patients who had undergone audiology evaluation, and for cochlear implantation, they were restricted to patients who had undergone candidacy evaluation. Statistical significance was set at *P* < 0.05, but given the large sample size, a strong signal of association was set as *P* < 0.001. Odds ratios are reported with 95% confidence intervals, formatted as (OR, point estimate; lower–upper bound).

## RESULTS

Among 1,461,489 US adults aged ≥65 years with bilateral SNHL in Epic Cosmos, 22,620 (1.55%) had a CI status code, and 15,415 (1.1%) received a CI candidacy evaluation during the study period. Both are cohort-wide counts, calculated against the full cohort rather than sequentially. CI status exceeds captured candidacy evaluations because implant status codes persist in the record regardless of when implantation occurred, whereas candidacy evaluation was counted only when billed during the study period. These totals are therefore distinct from the stage-specific pathway analysis as shown in Table 1, where each step is restricted to patients who completed the preceding step.

**TABLE 1.** Cochlear implant care pathway

Step in cochlear implant care pathway	N	%
Adults ≥65 years with bilateral SNHL	1,461,489	—
Received audiology evaluation	746,118	51.05% of patients ≥65 years with bilateral SNHL
Cochlear implant candidacy evaluation	9944	1.33% of those who received audiology evaluation
Cochlear implant surgery	9804	63.6% of those who received candidacy evaluation^a^

a Cochlear implant surgery percentage is calculated among all patients with a candidacy evaluation (n = 15,415), regardless of whether a prior audiological evaluation was captured, to reflect the candidacy-to-surgery transition.

SNHL indicates sensorineural hearing loss.

In Table 1 with stage-specific pathway analysis, 51.05% of the cohort received an audiology evaluation (n = 746,118); among those patients, 1.33% underwent CI candidacy evaluation (n = 9,944). Because some candidacy evaluations were not preceded by a captured audiology visit, the candidacy-to-surgery transition was assessed across the full CI candidacy population (n = 15,415, including 9,944 with a captured audiology evaluation), of whom 63.6% proceeded to surgery (n = 9,804). Table 2 summarizes demographic characteristics in each cohort.

**TABLE 2.** Cochlear implant care pathway among adults with bilateral SNHL

Characteristic	Base patient cohort (N = 1,461,489)	Audiology examination (N = 746,118)	Candidacy evaluation (N = 9,944)	Cochlear implant surgery (N = 9,804)
Sex				
Female	743,259 (51)	403,125 (54)	4190 (42)	4389 (45)
Male	718,129 (49)	342,954 (46)	5753 (58)	5415 (55)
None of the above	101 (0)	39 (0)	1 (0)	
Age				
≥65–<75	549,071 (38)	324,224 (43)	3124 (31)	3170 (32)
≥75–<85	565,499 (39)	287,106 (38)	4362 (44)	4259 (43)
85+	346,919 (24)	134,788 (18)	2458 (25)	2375 (24)
Race				
White	1,232,185 (84.31)	614,939 (82.42)	8756 (88.05)	8855 (90.32)
Black	111,337 (7.62)	68,956 (9.24)	160 (1.61)	463 (4.72)
Asian	51,589 (3.53)	26,114 (3.50)	244 (2.45)	214 (2.18)
Native Hawaiian/other PI	5265 (0.36)	2665 (0.36)	21 (0.21)	19 (0.19)
American Indian/Alaska Native	10,905 (0.75)	5841 (0.78)	79 (0.79)	64 (0.65)
Other	131,281 (8.98)	72,180 (9.67)	868 (8.73)	791 (8.07)
None of the above	45,337 (3.10)	24,261 (3.25)	242 (2.43)	211 (2.15)
Rural–urban community				
Metropolitan	1,223,718 (83.73)	629,337 (84.35)	8,010 (80.55)	7,838 (79.95)
Micropolitan	116,583 (7.98)	55,067 (7.38)	898 (9.03)	940 (9.59)
Small town/rural	107,021 (7.32)	55,258 (7.41)	939 (9.44)	931 (9.50)
N/A	14,167 (0.97)	6,456 (0.87)	97 (0.98)	95 (0.97)
Primary payer				
Medicaid	94,767 (6.48)	55,966 (7.50)	485 (4.88)	336 (3.43)
Medicare	1,254,324 (85.83)	644,721 (86.41)	8878 (89.28)	8795 (89.71)
Commercial	434,034 (29.70)	251,195 (33.67)	2760 (27.76)	2621 (26.73)
Self-pay	38,091 (2.61)	22,695 (3.04)	264 (2.65)	233 (2.38)
Not Insured	24,557 (1.68)	16,702 (2.24)	245 (2.46)	257 (2.62)
Other	79,863 (5.46)	37,196 (4.99)	475 (4.78)	471 (4.80)
None of the Above	53,691 (3.67)	12,910 (1.73)	195 (1.96)	211 (2.15)
US census region				
Midwest	449,302 (30.74)	238,322 (31.94)	3387 (34.06)	2954 (30.13)
Northeast	332,754 (22.77)	170,967 (22.91)	1700 (17.10)	1531 (15.62)
South	446,601 (30.56)	232,596 (31.17)	3404 (34.23)	3531 (36.02)
West	219,642 (15.03)	97,618 (13.08)	1369 (13.77)	1696 (17.30)
None of the above	13,190 (0.90)	6,615 (0.89)	84 (0.84)	92 (0.94)
Distance to cochlear implant center				
Within 25 miles	877,815 (60)	466,927 (62.58)	5819 (59)	5460 (56)
Within 25–50 miles	226,364 (15)	105,826 (14.18)	1651 (17)	1708 (17)
≥50 miles	253,080 (17.32)	127,679 (17.11)	1876 (18.9)	1957 (20)
None of the above	104,230 (7.13)	45,686 (6.12)	598 (6.01)	679 (6.93)
SVI tertile				
0–33% (lowest vulnerability)	605,743 (41)	99,918 (13)	3941 (40)	3926 (40)
33–66%	438,101 (30)	70,250 (9)	3087 (31)	3158 (32)
66–100%	400,446 (27)	51,995 (7)	2802 (28)	2611 (27)
None of the Above	17,199 (1.18)	2,769 (0.37)	114 (1.15)	109 (1.11)

SVI indicates Social Vulnerability Index.

**FIG. 1.**
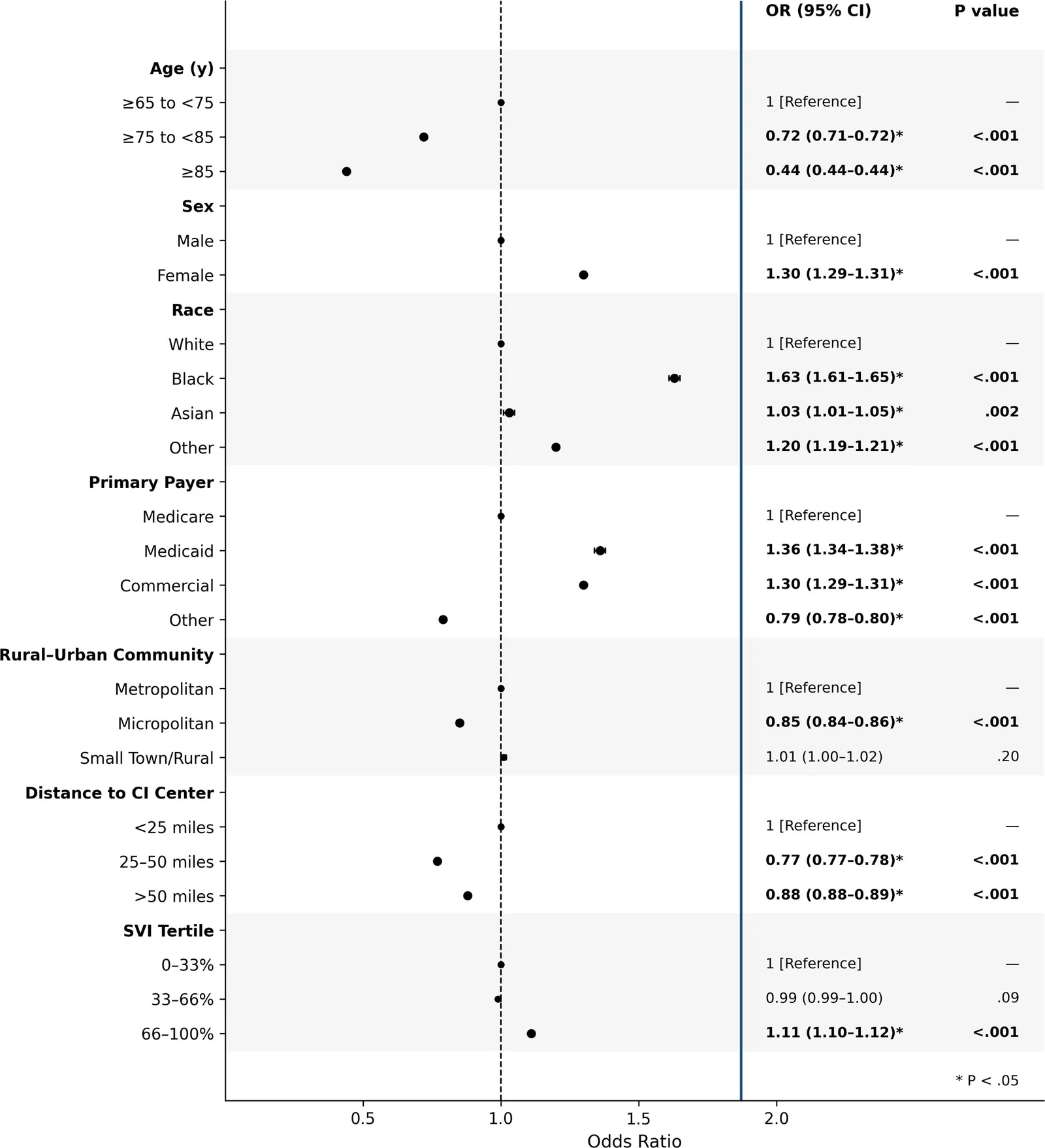
Odds ratios of a patient ≥65 years with bilateral sensorineural hearing loss receiving an audiology examination. OR indicates odds ratios; SVI, Social Vulnerability Index.

**FIG. 2.**
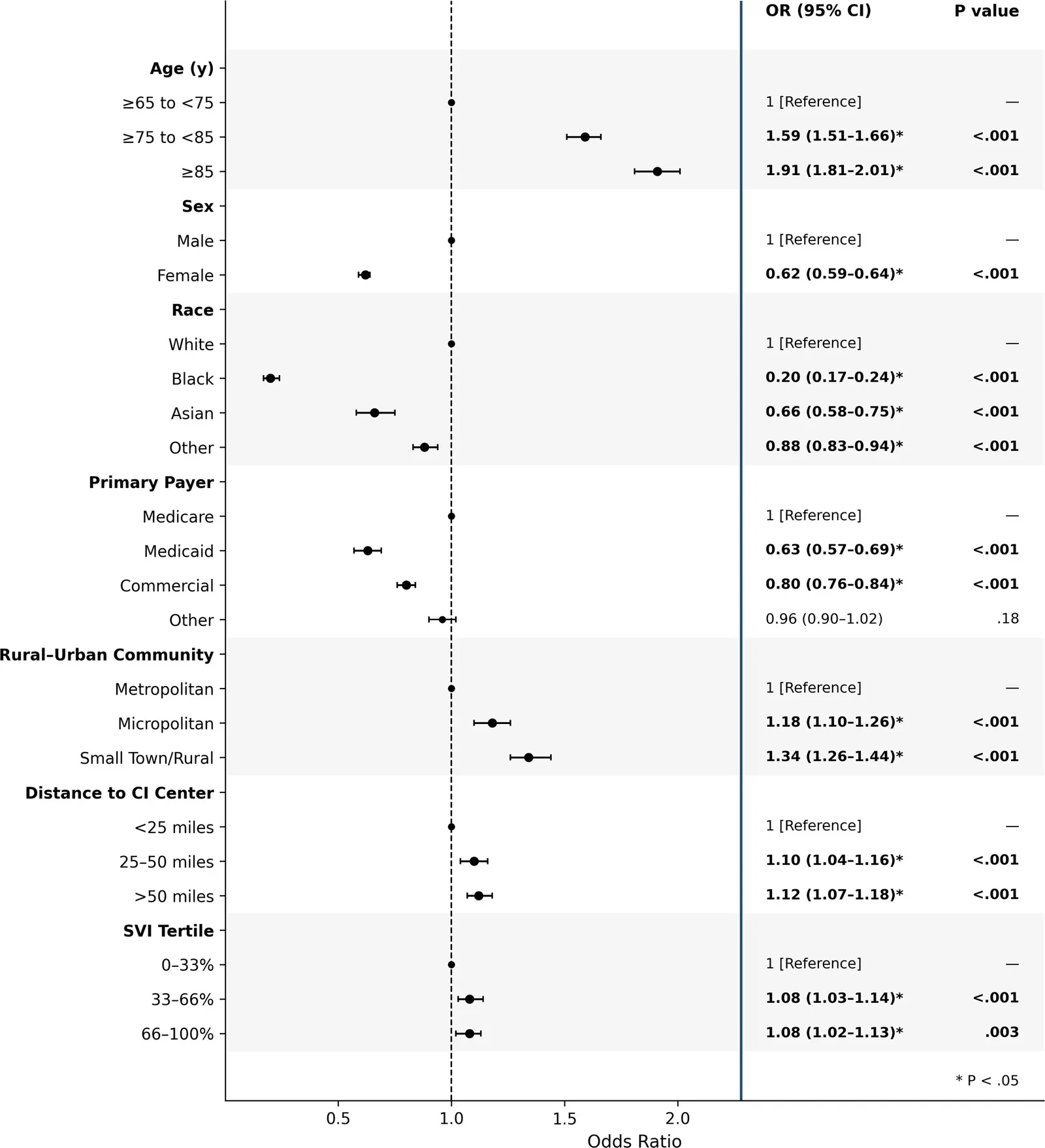
Odds ratios of a patient receiving a cochlear implant candidacy evaluation of those who received audiology evaluation. OR indicates odds ratios; SVI, Social Vulnerability Index.

**FIG. 3.**
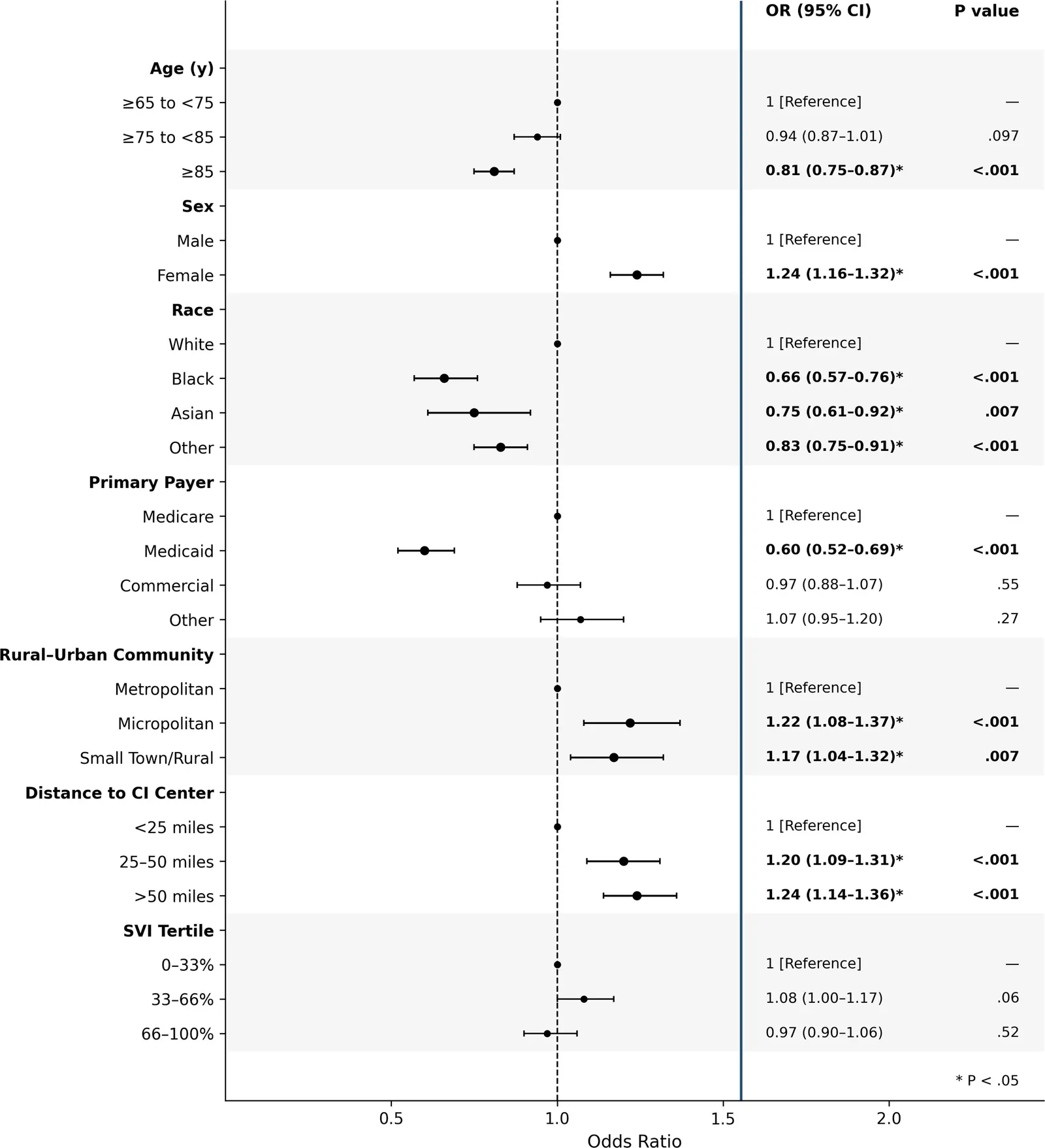
Odds ratios of a patient receiving a cochlear implant of those who had a cochlear implant candidacy evaluation. OR indicates odds ratios; SVI, Social Vulnerability Index.

Figure 1 illustrates the adjusted ORs for receiving an audiology evaluation among adults aged ≥65 years with bilateral SNHL. Figure 2 presents the adjusted ORs for undergoing CI candidacy evaluation among patients who completed an audiology evaluation. Figure 3 shows the adjusted ORs for receiving a CI among patients who underwent CI candidacy evaluation.

Compared with younger patients aged ≥65 to <75, older adults (age ≥85) were less likely to enter hearing care (OR, 0.44; 0.44–0.44, Fig. 1), more likely to be evaluated for CI candidacy once in care (OR, 1.91; 1.81–2.01, Fig. 2), but less likely to ultimately receive an implant (OR, 0.81; 0.75–0.87, Fig. 3). A similar but weaker association was seen when comparing adults aged ≥75 to <85 with those aged ≥65 to <75.

Women were more likely than men to undergo audiology evaluation (OR, 1.30; 1.29–1.31, Fig. 1) but were less likely to move from audiology to candidacy evaluation (OR, 0.62; 0.59–0.64, Fig. 2). Among those who did reach candidacy evaluation, this pattern reversed as women had higher odds of cochlear implantation (OR, 1.24; 1.16–1.32, Fig. 3).

Racial and ethnic disparities were most evident at the candidacy evaluation stage. Black and Asian patients cleared the first stage more readily than White patients, with higher odds of receiving an audiology evaluation (OR, 1.63; 1.61–1.65 and OR, 1.03; 1.01–1.05, respectively, in Fig. 1). Yet this early advantage reversed at every subsequent stage: both groups had significantly lower odds of CI candidacy evaluation (OR, 0.20; 0.17–0.24 and OR, 0.66; 0.58–0.75, Fig. 2) and, among those evaluated, of implantation (OR, 0.66; 0.57–0.76 and OR, 0.75; 0.61–0.92, Fig. 3).

Rurality demonstrated associations that varied in direction across the care continuum. Compared with metropolitan residents, individuals in micropolitan areas had lower odds of receiving audiological services (OR, 0.85; 0.84–0.86, Fig. 1), whereas micropolitan and rural residents had elevated odds of candidacy evaluation and cochlear implantation.

Distance to a CI surgical center showed an inverse association at the earliest stage of audiology services, with individuals living farther demonstrating reduced odds relative to those within 25 miles. In contrast, greater distance (>50 miles) was associated with elevated odds of having a CI candidacy evaluation (OR, 1.12; 1.07–1.18, Fig. 2) and CI surgery (OR, 1.24; 1.14–1.36, Fig. 3).

## DISCUSSION

In this large national analysis, disparities in CI access arise in a highly stage-specific manner across the care cascade. Using prior population-based National Health and Nutrition Examination Survey modeling, Yu et al (40) estimated that approximately 3.7% of US adults aged 65 years or older (~2.08 million individuals) meet expanded Centers for Medicare and Medicaid Services audiologic criteria for cochlear implantation in bilateral hearing loss.

In comparison, 22,620 of 1.46 million older adults with bilateral SNHL in this dataset (1.55%) had CI status documented. Although Epic Cosmos does not capture individual-level candidacy determinations, the discrepancy between modeled candidacy prevalence and observed implantation suggests that many older adults who could benefit from cochlear implantation remain unimplanted.

A consistent theme across sociodemographic and geographic factors was disengagement at key transition points in the care pathway, rather than a global disconnect from care. Advanced age (≥85 years) was associated with a stage-dependent pattern: lower odds of entering audiology care, higher odds of candidacy evaluation once evaluated, and lower odds of implantation thereafter. This locates the reduced CI utilization for the oldest adults at entry into care and at the surgical step rather than at candidacy determination. Interventions to increase hearing screening in older and rural populations, utilizing primary care-driven referrals or nonaudiology private hearing aid centers not captured in this dataset, may meaningfully influence downstream care engagement.

Geographic context shaped where along the pathway disparities emerged. Individuals in micropolitan and rural areas, and those living farther from a CI center, demonstrated lower odds of accessing audiological services, yet higher odds of progressing once engaged. This paradoxical pattern is consistent with a selection effect: patients who overcome the barrier of distance may differ systematically from those who do not, in terms of clinical severity, motivation, insurance coverage, or upstream referral filtering, such that those who reach care are more likely to progress through it.

Zhan et al (14) reported wide geographic variation in the United States and proposed under-referral after the audiology stage as a key contributor to low CI utilization, even in regions with high use of audiologic services. While this analysis does not assess temporal trends, the observed patterns in this study are consistent with referral inefficiencies early in the care pathway.

Age alone should not limit CI surgery since older adults, especially those 80 years and older, experience meaningful improvement in quality of life and speech following implantation (2,41,42). Barnes et al (43) demonstrated the importance of timely referral, showing that adult CI recipients present with speech perception well below current eligibility thresholds, a pattern that disproportionately affects older adults who also showed lower audiology utilization.

Prior single-center studies have shown that younger age and non-White race had higher odds of being deemed eligible for cochlear implantation, yet White patients were more likely to proceed to surgery (20,44,45). These observations are not necessarily inconsistent with the present findings, and apparent paradoxes have plausible explanations. In a single-center study of 823 adult evaluations (20), younger age was the only independent predictor of qualifying for implantation, with each additional year of age slightly lowering the odds of qualification; this likely reflects delayed referral, as older patients may present later in their hearing-loss trajectory, when comorbidities and other factors complicate candidacy.

The pattern by race is analogous. The higher candidacy rates among non-White patients likely reflect more severe hearing loss at the time of evaluation. Critically, these earlier studies characterized candidacy determination among patients who had already reached formal evaluation, whereas the present analysis characterizes progression to evaluation. Together, the lower odds of reaching evaluation among non-White patients and their higher conditional eligibility once evaluated suggest that disparities arise predominantly upstream, at referral and entry into evaluation, rather than at the point of clinical eligibility determination. Several mechanisms may contribute to this upstream gap: limited access to non-English CI testing batteries may underlie part of the association across races, and a scoping review of barriers to CI utilization has identified additional structural, societal, organizational, and interpersonal factors that could be targeted by intervention (46).

Taken together, these findings indicate that CI underutilization stems from gaps across the care pathway, rather than from a lack of access to hearing care alone. Because attrition was stage-specific, interventions are likely to be most effective when matched to the transition they target. At the entry stage, strengthening connections between primary care physicians, audiologists, and private hearing aid centers may improve patient education and facilitate earlier entry into the hearing care pathway. Telehealth may further mitigate geographic and distance-related barriers (47,48).

At the referral-to-candidacy transition, where attrition was steep, standardizing referral practices through validated clinical audiologic measures may reduce variability and ensure timely identification of candidates (49,50).

At the final treatment stage, expanding the pool of providers trained in cochlear implantation and increasing the geographic reach of CI programs may support equitable progression for patients who would meaningfully benefit from implantation (39).

## LIMITATIONS

This study draws on the scale and diversity of the Epic Cosmos dataset, providing a large and heterogeneous sample that represents hearing care patterns across US health systems, geographic regions, and patient populations (51–53). Because diagnoses and procedures are entered by treating clinicians, the data capture real-world clinical decision-making and care delivery. However, despite standardized quality controls, including Joint Commission compliance and internal auditing, variation in coding practices and incomplete or inaccurate documentation may affect the completeness and fidelity of the data and cannot be fully eliminated. In addition, the requirement of at least 2 encounters within a 2-year window, although intended to ensure longitudinal chart capture, may have preferentially excluded patients with the most limited healthcare access and could therefore lead the analysis to underestimate the magnitude of access-related disparities.

Epic Cosmos does not include audiometric threshold or speech recognition measures, which are essential for determining CI candidacy. Given that recent evidence shows CI eligibility can shift substantially depending on the speech criteria applied, candidacy cannot be accurately inferred from diagnostic coding alone; moreover, the dataset does not capture private or non-Epic hearing care systems (40). Thus, differences observed across groups could reflect both variations in candidacy qualification and access. Therefore, this study focuses on the utilization of evaluation and implantation services, rather than the determination of candidacy status.

Furthermore, Epic Cosmos provides aggregate rather than individual patient-level data, which prevents adjustment of the reported ORs. Because the aggregate structure precludes multivariable adjustment for comorbidity burden, functional status, and patient preference, observed trends (such as lower cochlear implantation rates in older populations) may be partially driven by factors that could not be accounted for, including medical comorbidities that increase surgical risk and reduce surgery rates. These findings should therefore be interpreted as population-level patterns rather than independent causal drivers, and some of the variation that appears to reflect differential access may instead stem from appropriate, individualized clinical decision-making rather than inequitable care.

ZIP codes are powerful and widely used indicators of community-level health context, but they are designed for mail delivery rather than spatial analysis and can vary in geographic size and population characteristics (54–56). As a result, distance estimates based on ZIP code centroids approximate potential geographic access, not actual travel burden. We supplemented this with RUCA codes and SVI to better capture structural and social context, though these measures also operate at the community level and may not fully reflect individual experience. Finally, the presence of a CI surgical center in proximity does not capture variation in actual access, such as appointment wait times, surgical scheduling frequency, or visiting-clinic coverage. Thus, proximity should be interpreted as potential geographic access rather than real-time availability of CI evaluation or surgery.

## CONCLUSION

In this national analysis, only 1.55% of elderly patients with bilateral SNHL had a documented CI. Disparities were stage-specific and group-dependent: among the oldest adults, they emerged at audiology evaluation and CI surgery; among non-White patients, gaps emerged at the transition to CI candidacy assessment and implantation, despite comparable or greater access to initial audiology. Because no single point of intervention can address barriers distributed across the pathway, improving CI utilization will require multifaceted, pathway-specific interventions, including patient education about hearing care resources, standardized referral processes, and expansion of the range and geographic reach of CI centers to ensure equitable access to hearing care options amid the rise of age-related hearing loss.

## ACKNOWLEDGMENTS

The authors would like to thank Bryan McConomy, Linda Owens, Emily Wee, Debby Vannoy, Pranav Dorbala, and Al Smith for their guidance and support throughout this work. The authors also acknowledge the Epic Cosmos team, Carle Illinois College of Medicine, and Carle Foundation Hospital for their collaboration and institutional support for student-led research.

## FUNDING SOURCE

None declared.

## CONFLICTS OF INTEREST STATEMENT

None declared.

## DATA AVAILABILITY STATEMENT

Data used in this study were obtained from Epic Cosmos, a limited dataset created in collaboration with a community of health systems using Epic, representing more than 300 million patient records from over 1,884 hospitals and 42,400 clinics as of February 2026. The community includes patients from all 50 US states, the District of Columbia, Canada, Lebanon, and Saudi Arabia. Current counts are available at cosmos.epic.com. Anonymized data are available through the Epic Cosmos platform in accordance with institutional data use policies at cosmos.epic.com.

## Supplementary Material



## References

[1] Zhu J Yang M Zhou C Kang H Wang D. Global burden and trends of age-related and other hearing loss: a 32-year analysis and future projections based on the GBD 2021. PLoS One. 2025;20:e0330690. 10.1371/journal.pone.0330690 PMC12370029 40839651

[2] Do BST Bush ML Weinreich HM. Clinical practice guideline: age-related hearing loss. Otolaryngol Head Neck Surg. 2024;170:S1–54. 10.1002/ohn.750 38687845

[3] Age-Related Hearing Loss (Presbycusis). 2023. Available at: https://www.nidcd.nih.gov/health/age-related-hearing-loss. Accessed October 27, 2025.

[4] Cantuaria ML Pedersen ER Waldorff FB. Hearing loss, hearing aid use, and risk of dementia in older adults. JAMA Otolaryngol Head Neck Surg. 2024;150:157–164. 10.1001/jamaoto.2023.3509 PMC10767640 38175662

[5] Tucker LH Grewal MR Chern A. Associations between hearing loss and dementia in a large electronic health record system. Laryngoscope. 2025;135:3840–3849. 10.1002/lary.32273 40384632

[6] Fu X Eikelboom RH Liu B Wang S Jayakody DMP. The impact of untreated hearing loss on depression, anxiety, stress, and loneliness in tonal language-speaking older adults in China. Front Psychol. 2022;13:917276. 10.3389/fpsyg.2022.917276 PMC9751871 36532984

[7] Foster JI Williams KL Timmer BHB Brauer SG. The association between hearing impairment and postural stability in older adults: a systematic review and meta-analysis. Trends Hear. 2022;26:23312165221144155. 10.1177/23312165221144155 PMC9761226 36524292

[8] Tan CJW Koh JWT Tan BKJ. Association between hearing loss and cardiovascular disease: a meta-analysis. Otolaryngol Head Neck Surg. 2024;170:694–707. 10.1002/ohn.599 38063267

[9] Zheng J Cheng Y Zhan Y Liu C Lu B Hu J. Cardiocerebrovascular risk in sensorineural hearing loss: results from the National Health and Nutrition Examination Survey 2015 to 2018. Front Neurol. 2023;14:1115252. 10.3389/fneur.2023.1115252 PMC10353435 37470009

[10] Cheng AK Niparko JK. Cost-utility of the cochlear implant in adults: a meta-analysis. Arch Otolaryngol Head Neck Surg. 1999;125:1214–1218. 10.1001/archotol.125.11.1214 10555692

[11] Benatti A Montino S Girasoli L. Cochlear implantation in the elderly: surgical and hearing outcomes. BMC Surg. 2013;13:S1. 10.1186/1471-2482-13-S2-S1 PMC3851201 24267394

[12] Andries E Gilles A Topsakal V. Systematic review of quality of life assessments after cochlear implantation in older adults. Audiol Neurootol. 2021;26:61–75. 10.1159/000508433 32653882

[13] Bourn SS Goldstein MR Morris SA Jacob A. Cochlear implant outcomes in the very elderly. Am J Otolaryngol. 2022;43:103200. 10.1016/j.amjoto.2021.103200 34600410

[14] Zhan KY Mazul A Kallogjeri DL Buchman CA. Use of diagnostic audiology and cochlear implantation in the US. JAMA Otolaryngol Head Neck Surg. 2024;150:353–354. 10.1001/jamaoto.2023.4738 PMC10884948 38386348

[15] Patel AA Levi JR Weber PC. Examining the impact of race, ethnicity, and language on pursuit of cochlear implantation. Clin Otolaryngol. 2025;51:25–31. 10.1111/coa.70020 40831433

[16] Reed NS Altan A Deal JA. Trends in health care costs and utilization associated with untreated hearing loss over 10 years. JAMA Otolaryngol Head Neck Surg. 2019;145:27–34. 10.1001/jamaoto.2018.2875 PMC6439810 30419131

[17] Zwolan TA Schvartz-Leyzac KC Pleasant T. Development of a 60/60 guideline for referring adults for a traditional cochlear implant candidacy evaluation. Otol Neurotol. 2020;41:895–900. 10.1097/MAO.0000000000002664 32658396

[18] Zeitler DM Prentiss SM Sydlowski SA Dunn CC. American cochlear implant alliance task force: recommendations for determining cochlear implant candidacy in adults. Laryngoscope. 2024;134:S1–S14. 10.1002/lary.30879 PMC10914083 37435829

[19] Quimby AE Venkatesh S Corsten M. Socioeconomic status among cochlear implant candidates and association with surgical pursuance. JAMA Otolaryngol Head Neck Surg. 2023;149:891–898. 10.1001/jamaoto.2023.2217 PMC10450586 37615991

[20] Tolisano AM Schauwecker N Baumgart B. Identifying disadvantaged groups for cochlear implantation: demographics from a large cochlear implant program. Ann Otol Rhinol Laryngol. 2020;129:347–354. 10.1177/0003489419888232 31735055

[21] Tarabichi Y Frees A Honeywell S. The cosmos collaborative: a vendor-facilitated electronic health record data aggregation platform. ACI open. 2021;5:e36–e46. 10.1055/s-0041-1731004 PMC8775787 35071993

[22] Gunderson MA Dorr DA Freedman H Melton GB. A longitudinal analysis of institutional adoption, use, and dissemination of an EHR vendor-based data sharing program. Stud Health Technol Inform. 2025;329:154–158. 10.3233/SHTI250820 40775838

[23] von Elm E Altman DG Egger M. The Strengthening the Reporting of Observational Studies in Epidemiology (STROBE) statement: guidelines for reporting observational studies. J Clin Epidemiol. 2008;61:344–349. 10.1016/j.jclinepi.2007.11.008 18313558

[24] Miyagishima R Hopper T Hodgetts B Soos B Williamson T Drummond N. Development of a case definition for hearing loss in community-based older adults: a cross-sectional validation study. CMAJ Open. 2021;9:E796–E801. 10.9778/cmajo.20200267 PMC8373040 34404687

[25] Deal JA Reed NS Kravetz AD. Incident hearing loss and comorbidity: a longitudinal administrative claims study. JAMA Otolaryngol Head Neck Surg. 2019;145:36–43. 10.1001/jamaoto.2018.2876 PMC6439817 30419134

[26] Pittman CA Roura R Price C Lin FR Marrone N Nieman CL. Racial/ethnic and sex representation in US-based clinical trials of hearing loss management in adults: a systematic review. JAMA Otolaryngol Head Neck Surg. 2021;147:656–662. 10.1001/jamaoto.2021.0550 33885733

[27] Garber S Ridgely MS Bradley M Chin KW. Payment under public and private insurance and access to cochlear implants. Arch Otolaryngol Head Neck Surg. 2002;128:1145–1152. 10.1001/archotol.128.10.1145 12365885

[28] Shi JJ Fujiwara RJT Punnen LM Sakano H Isaacson B. Decreasing medicare reimbursement for facility-performed neurotology procedures from 2000 to 2024. Otol Neurotol. 2025;46:871–876. 10.1097/MAO.0000000000004524 40466113

[29] Fujiwara RJT Wong EC Ishiyama A. Geographic variations in medicare cochlear implantations in the United States. Otol Neurotol. 2022;43:1022–1026. 10.1097/MAO.0000000000003660 36006783

[30] Hambach B Patel J Nunes K. Impact of implantable hearing devices on delirium risk in patients with hearing loss: a national database study. Otol Neurotol. 2025;46:775–780. 10.1097/MAO.0000000000004416 40059784

[31] Saunders GH Dillard LK Zobay O Cannon JB Naylor G. Electronic health records as a platform for audiological research: data validity, patient characteristics, and hearing-aid use persistence among 731,213 U.S. veterans. Ear Hear. 2021;42:927–940. 10.1097/AUD.0000000000000980 PMC8221720 33974367

[32] Qian ZJ Chang KW Ahmad IN Tribble MS Cheng AG. Use of diagnostic testing and intervention for sensorineural hearing loss in US children from 2008 to 2018. JAMA Otolaryngol Head Neck Surg. 2021;147:253–260. 10.1001/jamaoto.2020.5030 PMC7774052 33377936

[33] Friedman HR Holmes GM. Rural Medicare beneficiaries are increasingly likely to be admitted to urban hospitals. Health Serv Res. 2022;57:1029–1034. 10.1111/1475-6773.14017 PMC9441274 35773787

[34] Adams MCB Hudson CL Perkins ML Hurley RW Topaloglu U. Leveraging the rural-urban commuting area tool to address geographic disparities in cancer care: a dual-application framework for institutional and national initiatives. JCO Clin Cancer Inform. 2025;9:e2500122. 10.1200/CCI-25-00122 PMC12614380 41202192

[35] Hung P Probst JC Shih Y. Rural-urban disparities in quality of inpatient psychiatric care. Psychiatr Serv. 2023;74:446–454. 10.1176/appi.ps.20220277 36321319

[36] Torres-Small S Ward CN Thurmond SL. Inclusive soundscapes: how race, socioeconomic status and maternal age influence the pediatric cochlear implant journey. Otolaryngol Head Neck Surg. 2025;172:651–660. 10.1002/ohn.1024 39413347

[37] Qian ZJ Rehkopf DH. Association between social disadvantage and otitis media treatment in US children with commercial insurance. JAMA Otolaryngol Head Neck Surg. 2022;149:7–14. 10.1001/jamaoto.2022.3560 PMC9650625 36355356

[38] Desai P Bond J Dhana A. The social vulnerability index and incidence of alzheimer disease in a population-based sample of older adults. Neurology. 2025;104:e213464. 10.1212/WNL.0000000000213464 PMC11962047 40138617

[39] Espahbodi M Trautwein P Bestourous DE. Access to cochlear implantation: trends in surgeon volume and training. Laryngoscope. 2025;135:2146–2153. 10.1002/lary.32037 PMC12081994 39932075

[40] Yu K Shen S Bowditch S Sun D. Estimating the United States patient population size meeting audiologic candidacy for cochlear implantation. Otolaryngol Head Neck Surg. 2024;170:870–876. 10.1002/ohn.589 PMC10922682 37997296

[41] Zwolan TA Kallogjeri D Firszt JB Buchman CA. Assessment of cochlear implants for adult medicare beneficiaries aged 65 years or older who meet expanded indications of open-set sentence recognition. JAMA Otolaryngol Head Neck Surg. 2020;146:933–939. 10.1001/jamaoto.2020.2286 PMC7453340 32857106

[42] Buchman CA Gifford RH Haynes DS. Unilateral cochlear implants for severe, profound, or moderate sloping to profound bilateral sensorineural hearing loss: a systematic review and consensus statements. JAMA Otolaryngol Head Neck Surg. 2020;146:942–953. 10.1001/jamaoto.2020.0998 32857157

[43] Barnes JH Yin LX Marinelli JP Carlson ML. Audiometric profile of cochlear implant recipients demonstrates need for revising insurance coverage. Laryngoscope. 2021;131:E2007. 10.1002/lary.29334 33347621

[44] Patro A Lindquist NR Tawfik KO. A five-year update on the profile of adults undergoing cochlear implant evaluation and surgery-are we doing better? Otol Neurotol. 2022;43:e992–e999. 10.1097/MAO.0000000000003670 36047696

[45] Mahendran GN Rosenbluth T Featherstone M Vivas EX Mattox DE Hobson CE. Racial disparities in adult cochlear implantation. Otolaryngol Head Neck Surg. 2022;166:1099–1105. 10.1177/01945998211027340 34311626

[46] Neukam JD Kunnath AJ Patro A. Barriers to cochlear implant uptake in adults: a scoping review. Otol Neurotol. 2024;45:e679–e686. 10.1097/MAO.0000000000004340 39514420

[47] Fletcher KT Dicken FW Adkins MM. Audiology telemedicine evaluations: potential expanded applications. Otolaryngol Head Neck Surg. 2019;161:63–66. 10.1177/0194599819835541 PMC6602851 30832542

[48] Frisby C Eikelboom R Mahomed-Asmail F Kuper H Swanepoel DW. m-Health applications for hearing loss: a scoping review. Telemed J E Health. 2022;28:1090–1099. 10.1089/tmj.2021.0460 34967683

[49] Prentiss S Snapp H Zwolan T. Audiology practices in the preoperative evaluation and management of adult cochlear implant candidates. JAMA Otolaryngol Head Neck Surg. 2020;146:136–142. 10.1001/jamaoto.2019.3760 PMC6990940 31830215

[50] Reddy P Dornhoffer JR Camposeo EL Dubno JR McRackan TR. Using clinical audiologic measures to determine cochlear implant candidacy. Audiol Neurootol. 2022;27:235–242. 10.1159/000520077 PMC9133005 35038700

[51] Hong CJ Kaur MN Farrokhyar F Thoma A. Accuracy and completeness of electronic medical records obtained from referring physicians in a Hamilton, Ontario, plastic surgery practice: a prospective feasibility study. Plast Surg Oakv Ont. 2015;23:48–50. 10.4172/plastic-surgery.1000900 PMC4364140 25821774

[52] Mankowski MA Bae S Strauss AT. Generalizability of kidney transplant data in electronic health records - the Epic Cosmos database vs the scientific registry of transplant recipients. Am J Transplant. 2025;25:744–755. 10.1016/j.ajt.2024.11.008 PMC11972892 39550008

[53] Goldstein ND Olivieri-Mui B Burstyn I. Are aggregated electronic health record datasets good for research? J Gen Intern Med. 2025;40:3743–3749. 10.1007/s11606-025-09808-9 PMC12612337 40794368

[54] Cooper RA Cooper MA McGinley EL Fan X Rosenthal JT. Poverty, wealth, and health care utilization: a geographic assessment. J Urban Health. 2012;89:828–847. 10.1007/s11524-012-9689-3 PMC3462827 22566148

[55] Benavidez GA Zahnd WE Hung P Eberth JM. Chronic disease prevalence in the US: sociodemographic and geographic variations by zip code tabulation area. Prev Chronic Dis. 2024;21:E14. 10.5888/pcd21.230267 PMC10944638 38426538

[56] Baum A Wisnivesky J Basu S Siu AL Schwartz MD. Association of geographic differences in prevalence of uncontrolled chronic conditions with changes in individuals’ likelihood of uncontrolled chronic conditions. JAMA. 2020;324:1429–1438. 10.1001/jama.2020.14381 PMC8094427 33048153

