# Supplementary material for: Access and Attrition Across Audiology and Cochlear Implant Care Among Older Adults with Bilateral Sensorineural Hearing Loss
Source: Otol Neurotol Open. 2026 Aug 11;6(3):e096. doi: 10.1097/ONO.0000000000000096 (PMC13610227; doi:10.1097/ONO.0000000000000096)
Supplement: Supplementary file 1 [file ono-6-e096-s001.pdf]

Supplemental Table 1. International Classification of Diseases (ICD)-10 codes used to identify exclusionary causes of hearing loss

Supplemental Table 1. International Classification of Diseases (ICD)-10 codes used to identify exclusionary causes of hearing loss

Category	ICD-10-CM	Description
Conductive and Mixed Hearing Loss (Non-Sensorineural Etiologies)	H90.0*	Conductive hearing loss, bilateral
	H90.1*	Conductive hearing loss, unilateral (with normal contralateral ear)
	H90.2*	Conductive hearing loss, unspecified
	H90.6*	Mixed conductive & sensorineural hearing loss, bilateral
	H90.8*	Mixed conductive & sensorineural hearing loss, unspecified
Sudden or Fluctuating Sensorineural Hearing Loss	H91.2*	Sudden idiopathic sensorineural hearing loss (all laterality)
	H81.0*	Ménière’s disease, specified ear
Otologic Symptoms / Secondary Conditions (Non-HL Etiologies)	H93.23*	Hyperacusis
	H92.1*	Otorrhea
	H92.0*	Otalgia
Unilateral / Asymmetric Sensorineural Hearing Loss	H90.4*	Sensorineural hearing loss, unilateral with unrestricted hearing on the contralateral side
Congenital or Surgically Correctable Conditions	Q16.*	Congenital malformations of ear causing impairment of hearing
	H80.*	Otosclerosis
	H71.*	Cholesteatoma of middle ear
	H65.*	Chronic nonsuppurative otitis media
	H66.*	Suppurative & unspecified otitis media
	H70.*	Mastoiditis & related conditions
Retro cochlear and Central Auditory Disorders	D33.3	Vestibular schwannoma / acoustic neuroma (benign cranial nerve neoplasm)
	C72.4*	Acoustic nerve malignancy
	H93.3X*	Disorders of acoustic nerve

	H93.25*	Central auditory processing disorder
Acute or Structural Inner-Ear Disorders (Labyrinthine Lesions)	H83.01	Labyrinthitis, right ear
	H83.02	Labyrinthitis, left ear
	H83.03	Labyrinthitis, bilateral
	H83.09	Labyrinthitis, unspecified ear
	H83.1*	Labyrinthine fistula
	H83.2*	Labyrinthine dysfunction /other specified labyrinthine disorders
Other Specified / Unclassified Inner-Ear Diseases	H83.8X1	Other specified diseases of right inner ear
	H83.8X2	Other specified diseases of left inner ear
	H83.8X3	Other specified diseases of inner ear, bilateral
Ototoxic Hearing Loss	H91.03	Ototoxic hearing loss, bilateral
	H91.02	Ototoxic hearing loss, left ear
	H91.01	Ototoxic hearing loss, right ear
	H91.09	Ototoxic hearing loss, unspecified ear
