# Supplementary material for: Access and Attrition Across Audiology and Cochlear Implant Care Among Older Adults with Bilateral Sensorineural Hearing Loss
Source: Otol Neurotol Open. 2026 Aug 11;6(3):e096. doi: 10.1097/ONO.0000000000000096 (PMC13610227; doi:10.1097/ONO.0000000000000096)
Supplement: Supplementary file 2 [file ono-6-e096-s002.pdf]

Supplemental Table 2. ICD-10 and Medical billing codes to identify patients with cochlear implant

Category	Type	Code	Description
Cochlear Implant	ICD-10-CM:	Z96.21	Cochlear implant status
		Z45.321	Encounter for adjustment & management of CI
	HCPCS	L8614	Cochlear device, complete system
	CPT	69930	Cochlear device implantation, w/ or w/o mastoidectomy
		92603	Post-operative analysis, fitting, and adjustments analysis of cochlear implant, with programming; 7 yrs or older
		92604	Post-operative analysis, fitting, and adjustments analysis of cochlear implant, subsequent reprogramming; 7 yrs or older
