# Supplementary material for: Access and Attrition Across Audiology and Cochlear Implant Care Among Older Adults with Bilateral Sensorineural Hearing Loss
Source: Otol Neurotol Open. 2026 Aug 11;6(3):e096. doi: 10.1097/ONO.0000000000000096 (PMC13610227; doi:10.1097/ONO.0000000000000096)
Supplement: Supplementary file 3 [file ono-6-e096-s003.pdf]

Supplemental Table 3. Medical billing codes to identify audiology or cochlear implant candidacy examination

Category	Type	Code	Description
Audiologic Evaluation	CPT	92550	Tympanometry and threshold measurements
		92553	Air bone audiometry
		92555	Speech audiometry threshold
		92556	Speech audiometry with speech recognition
		92557	Comprehensive audiometry threshold evaluation and speech recognition
		92567	Tympanometry
		92579	Visual reinforcement audiometry
		92585	Diagnostic auditory brain response
		92587	Distortion product otoacoustic emission[PPV1]
Cochlear Implant Candidacy Evaluation		92626	Evaluation of auditory rehabilitation status
