# Supplementary material for: Access and Attrition Across Audiology and Cochlear Implant Care Among Older Adults with Bilateral Sensorineural Hearing Loss
Source: Otol Neurotol Open. 2026 Aug 11;6(3):e096. doi: 10.1097/ONO.0000000000000096 (PMC13610227; doi:10.1097/ONO.0000000000000096)
Supplement: Supplementary file 4 [file ono-6-e096-s004.pdf]

Supplemental Table 4. Cochlear Implant Center List

| Name | Street Address | City | State | Zip Code |
|---|---|---|---|---|
| Alaska Center for Ear, Nose & Throat | 3801 University Lake Dr | Anchorage | AK | 99508 |
| Alabama Ear | 2937 7th Avenue South | Birmingham | AL | 35233 |
| ENT Associates of Alabama | 3686 Grandview Parkway | Birmingham | AL | 35243 |
| Arkansas Otolaryngology | 10201 Kanis Rd | Little Rock | AR | 72205 |
| VA Central AR Veterans Health Care System | 4300 W 7th St | Little Rock | AR | 72205 |
| UAMS Medical Center - ENT | 501 S Jack Stephens Dr | Little Rock | AR | 72205 |
| Mercy Northwest Arkansas ENT | 5204 W Redbud | Rogers | AR | 72758 |
| Banner Desert Medical Center | 1400 S Dobson Rd | Mesa | AZ | 85202 |
| Banner Univ Medical Center – Phoenix | 1111 E McDowell Rd | Phoenix | AZ | 85006 |
| Mayo Clinic Hospital – Phoenix | 5711 E Mayo Blvd | Phoenix | AZ | 85054 |
| St. Joseph’s Outpatient (Phoenix) | 240 W Thomas Rd | Phoenix | AZ | 85013 |
| Banner – Univ Med Center Tucson | 1625 N Campbell Ave | Tucson | AZ | 85719 |
| Tucson Medical Center | 5301 E Grant Rd | Tucson | AZ | 85712 |
| Kaiser Permanente Anaheim | 3440 E La Palma Ave | Anaheim | CA | 92806 |
| Kaiser Antioch | 4501 Sand Creek Rd | Antioch | CA | 94531 |
| Providence St Joseph Medical Center | 501 S Buena Vista St | Burbank | CA | 91505 |
| CALHNS Carlsbad | 2390 Faraday Ave | Carlsbad | CA | 92008 |
| CALHNS Chula Vista | 577 Third Ave | Chula Vista | CA | 91910 |
| Sharp Coronado Hospital | 250 Prospect Place | Coronado | CA | 92118 |
| Sharp Memorial Hospital | 7901 Frost St. San | Diego | CA | 92123 |
| UCSD Perlman Clinic (H&N Surg – Audiology) | 9350 Campus Point San | Diego | CA | 92037 |

| | | | | |
|---|---|---|---|---|
| Palo Alto Medical Foundation – Fremont | 3200 Kearney St | Fremont | CA | 94538 |
| UCI Medical Center | 2346 E Walnut Ave | Fullerton | CA | 92831 |
| San Diego ENT | 4150 Regents Park Row | La Jolla | CA | 92037 |
| Sharp Grossmont Hospital | 5555 Grossmont Center Drive | La Mesa | CA | 91942 |
| Loma Linda Univ Outpatient Surgery Ctr | 11370 Anderson St | Loma Linda | CA | 92354 |
| VA Loma Linda Medical Center | 11201 Benton St | Loma Linda | CA | 92357 |
| VA Healthcare System (Long Beach) | 5901 E 7th St | Long Beach | CA | 90822 |
| Cedars-Sinai Medical Center | 8700 Beverly Blvd | Los Angeles | CA | 90048 |
| Good Samaritan Hospital | 616 Witmer St | Los Angeles | CA | 90017 |
| Kaiser Permanente Sunset | 1550 N Edgemont St | Los Angeles | CA | 90027 |
| Pacific Neuroscience Institute | 11645 Wilshire Blvd | Los Angeles | CA | 90025 |
| PIH House Clinic (LA) | 1245 Wilshire Blvd | Los Angeles | CA | 90017 |
| UCLA Otology & Neurotology | 200 UCLA Medical Plaza | Los Angeles | CA | 90095 |
| USC Caruso Family Center | 1640 Marengo St | Los Angeles | CA | 90033 |
| USC University Hospital | 1500 San Pablo St | Los Angeles | CA | 90033 |
| Cedars-Sinai Marina del Rey | 4650 Lincoln Blvd | Marina del Rey | CA | 90292 |
| VA Med Ctr Martinez OPC | 150 Muir Rd | Martinez | CA | 94553 |
| VA Med Ctr Mather | 10535 Hospital Way | Mather | CA | 95655 |
| United ENT – Murrieta | 35100 Makena Ave | Murrieta | CA | 92563 |
| Hoag Memorial Hospital Presbyterian | 1 Hoag Dr | Newport Beach | CA | 92658 |
| Shohet Ear Associates Medical Group | 446 Old Newport Blvd | Newport Beach | CA | 92663 |
| Kaiser Permanente Oakland | 3600 Broadway | Oakland | CA | 94611 |
| La Veta Surgical Center | 681 S Parker St | Orange | CA | 92868 |

| | | | | |
|---|---|---|---|---|
| PIH Health House Clinic – Orange County | 431 S Batavia St | Orange | CA | 92868 |
| Rady Restorative Care Center | 1301 Providence Ave | Orange | CA | 92868 |
| UCI Medical Center (Pavilion II H&N) | 101 The City Dr. | Orange | CA | 92868 |
| Kaiser Permanente Riverside | 10800 Magnolia Ave | Riverside | CA | 92505 |
| Kaiser South Sacramento | 7300 Wyndham Dr | Sacramento | CA | 95823 |
| Sacramento ENT | 1111 Exposition Blvd | Sacramento | CA | 95815 |
| UC Davis Medical Center | 2521 Stockton Blvd | Sacramento | CA | 95817 |
| CALHNS Sorrento Valley | 5405 Oberlin Dr | San Diego | CA | 92121 |
| Kaiser Permanente Copley/Garfield | 5893 Copley Dr | San Diego | CA | 92111 |
| Scripps Rancho Bernardo | 15004 Innovation Dr | San Diego | CA | 92128 |
| UCSD Hillcrest | 200 W Arbor Dr | San Diego | CA | 92103 |
| Sea Surgery Center, LLC | 770 Pacific Coast Hwy | Seal Beach | CA | 90740 |
| United ENT – Temecula | 44605 Avenida De Missiones | Temecula | CA | 92592 |
| Tahoe Forest Hospital | 10121 Pine Ave | Truckee | CA | 96161 |
| Tahoe Forest MultiSpecialty Clinic | 12313 Soaring Way | Truckee | CA | 96161 |
| Sharp Chula Vista Medical Center | 751 Medical Center Ct | Chula Vista | CA | 91911 |
| Kaiser Permanente Woodland Hills | 5601 De Soto Ave | Woodland Hills | CA | 91367 |
| UCHealth Ear, Nose and Throat Clinic - Anschutz Medical Campus | 1635 Aurora Court | Aurora | CO | 80045 |
| UCHealth Ear, Nose and Throat Clinic - Eastview | 3843 Rio Vista Drive | Colorado Springs | CO | 80917 |
| Colorado ENT & Allergy Audubon Medical Campus | 3030 N. Circle Dr. | Colorado Springs | CO | 80909 |
| COLORADO SPRINGS EAR ASSOCIATES | 2950 Professional Place | Colorado Springs | CO | 80904 |
| UCHealth Cherry Creek Medical Center | 100 Cook Street | Denver | CO | 80206 |
| Denver Ear Associates | 701 E Hampden Ave | Englewood | CO | 80113 |

| | | | | |
|---|---|---|---|---|
| Alpine ENT | 1120 E Elizabeth St | Fort Collins | CO | 80524 |
| UCHealth Ear, Nose and Throat Clinic - Mason Street | 4601 S. Mason Street | Fort Collins | CO | 80525 |
| UCHealth Ear, Nose and Throat Clinic - Lone Tree | 9548 Park Meadows Drive | Lone Tree | CO | 80124 |
| BMC Longmont | 1551 Professional Lane | Longmont | CO | 80501 |
| BMC Louisville | 80 Health Park Dr. | Louisville | CO | 80027 |
| Univ of Connecticut Health Center | 263 Farmington Ave | Farmington | CT | 6030 |
| Hartford Hospital | 80 Seymour St | Hartford | CT | 6106 |
| Yale New Haven Hospital | 726 Howard Ave | New Haven | CT | 6519 |
| River Valley Ambulatory Surgery Center | 45 Salem Turnpike | Norwich | CT | 6360 |
| Stamford Hospital | One Hospital Plaza | Stamford | CT | 6902 |
| VA Med Ctr – West Haven | 950 Campbell Ave | West Haven | CT | 6516 |
| MedStar Georgetown Univ Hospital | 2115 Wisconsin Ave NW | Washington | DC | 20007 |
| MedStar Washington Hospital Center | 110 Irving Street NW | Washington | DC | 20010 |
| VA Medical Center – DC | 50 Irving St | Washington | DC | 20422 |
| Bayhealth Cochlear Implant Clinic | 101 Wellness Way | Milford | DE | 19963 |
| ENT and Allergy of Delaware LLC | 700 Prides Crossing | Newark | DE | 19713 |
| Jupiter Medical Center | 210 Jupiter Lakes Blvd | Jupiter | FL | 33458 |
| Mark Widick, MD - ENTAAF Delray | 5130 Linton Blvd | Beach | FL | 33484 |
| AdventHealth Medical Group | 410 Celebration Place | Celebration | FL | 34747 |
| UF Health Audiology at The Oaks | 6201 W Newberry Rd | Gainesville | FL | 32605 |
| S FL ENT Associates - Hollywood | 3800 Johnson St | Hollywood | FL | 33021 |
| Jacksonville Hearing & Balance Institute | 10475 Centurion Pkwy N | Jacksonville | FL | 32256 |
| Univ of Miami Sch of Med - Main | 1120 NW 14th Street | Miami | FL | 33136 |

| | | | | |
|---|---|---|---|---|
| VA Med Ctr Miami | 1201 NW 16th St | Miami | FL | 33125 |
| The Hearing and Balance Center – Orlando | 44 W Michigan St | Orlando | FL | 32806 |
| Baptist Medical Group ENT & Audiology | 125 Baptist Way | Pensacola | FL | 32501 |
| The Cochlear Clinic | 6540 4th Street North | St. Petersburg | FL | 33702 |
| VA Med Ctr Miami - Broward | 9800 West Commercial Blvd | Sunrise | FL | 33351 |
| ENT and Allergy Associates of Florida LLC | 5 Tampa General Circle | Tampa | FL | 33606 |
| USF Health Department of Otolaryngology - Head and Neck Surgery | 13330 Usf Laurel Drive | Tampa | FL | 33612 |
| The Hearing and Balance Center – Winter Park | 133 Benmore Drive | Winter Park | FL | 32792 |
| Atlanta Institute for ENT | 5670 Peachtree Dunwoody Rd | Atlanta | GA | 30342 |
| Emory Univ Hospital – Midtown | 550 Peachtree St NE | Atlanta | GA | 30308 |
| Piedmont ENT Hearing LLC | 1720 Peachtree St | Atlanta | GA | 30309 |
| Wellstar MCG Health – Audiology | 1120 15th Street | Augusta | GA | 30912 |
| WellStar Medical Group – Kennestone | 699 Church St. | Marietta | GA | 30060 |
| Pali Momi Medical Center | 98-1079 Moanalua Rd | Aiea | HI | 96701 |
| Kapiolani Medical Center for Women and Children | 1319 Punahou Street | Honolulu | HI | 96826 |
| Queen's Medical Center | 1301 Punchbowl Street | Honolulu | HI | 96813 |
| Kaiser Permanente Hawaii Moanalua | 3288 Moanalua Rd | Honolulu | HI | 96819 |
| Mercy Ear, Nose and Throat Clinic | 901 8th Ave SE | Cedar Rapids | IA | 52401 |
| Iowa Ear Center | 12499 University Avenue | Clive | IA | 50325 |
| Univ of Iowa Hospitals & Clinics | 200 Hawkins Dr | Iowa City | IA | 52242 |
| Iowa ENT Center | 105 Valley W Dr | West Des Moines | IA | 50265 |
| Southwest Idaho Surgery Center | 900 N Liberty | Boise | ID | 83704 |

| | | | | |
|---|---|---|---|---|
| Carle Foundation | 3105 Fields South Dr | Champaign | IL | 61822 |
| Northwestern Medicine Department of Otolaryngology | 675 N St Clair St | Chicago | IL | 60611 |
| Rush Univ Medical Center | 1611 W Harrison St, | Chicago | IL | 60612 |
| Univ of Chicago Hospitals | 5841 S Maryland Ave | Chicago | IL | 60637 |
| Univ of Illinois–Chicago | 1855 W Taylor St | Chicago | IL | 60612 |
| Northwestern Medicine Primary & Specialty Care Evanston | 1630 Sherman Ave | Evanston | IL | 60201 |
| VA Hospital – Edward Hines Jr. | 5000 S Fifth Ave | Hines | IL | 60141 |
| ENT Surgical Consultants | 2201 Glenwood Ave | Joliet | IL | 60435 |
| Loyola University Medical Center | 2160 S 1st Ave | Maywood | IL | 60153 |
| Rush OakBrook | 2011 York Rd, 2nd Floor | Oak Brook | IL | 60523 |
| Univ. of Chicago Ctr for Advanced Care | 14290 S. LaGrange Road | Orland Park | IL | 60462 |
| OSF HealthCare Illinois Neurological Ins | 200 E Pennsylvania Ave | Peoria | IL | 61603 |
| SIU Clinic for Hearing & Balance | 720 N Bond St. | Springfield | IL | 62702 |
| Central Dupage Hospital | 25 N Winfield Rd | Winfield | IL | 60190 |
| Otolaryngology Associates (Carmel) | 10201 Illinois Street | Carmel | IN | 46032 |
| Ear, Nose & Throat Associates, P.C. | 10021 Dupont Circle Ct | Fort Wayne | IN | 46825 |
| Indiana Ear | 9604 Coldwater Rd | Fort Wayne | IN | 46825 |
| Indiana Univ Fort Wayne Outpatient | 4105 Dicke Rd | Fort Wayne | IN | 46804 |
| Otolaryngology Associates of NWI | 2211 Main St | Highland | IN | 46322 |
| Indiana University – Cochlear Implant Program | 705 Riley Hospital Drive | Indianapolis | IN | 46202 |
| Franciscan Physician Network | 1225 E Coolspring Ave | Michigan City | IN | 46360 |
| UChicago Medicine Crown Point | 10855 Virginia St. | Crown Point | IN | 46307 |
| Community Neuroscience & Sports Medicine | 1545 W US Highway 30 | Schererville | IN | 46375 |

| | | | | |
|---|---|---|---|---|
| The University of Kansas Hospital | 4000 Cambridge Street | Kansas City | KS | 66160 |
| Midwestern Ear, Nose, & Throat - Kansas | 20375 West 151st Street | Olathe | KS | 66061 |
| Medical Pavillion | 10730 Nall Avenue | Overland Park | KS | 66211 |
| KU MedWest | 7405 Renner Road | Shawnee | KS | 66217 |
| Univ of Kentucky Medical Center | 740 S Limestone St, | Lexington | KY | 40536 |
| Univ of Louisville Physicians | 401 East Chestnut St | Louisville | KY | 40202 |
| Acadian ENT | 1000 W Pinhook Rd | Lafayette | LA | 70503 |
| Camellia ENT | 1039 Camellia Blvd | Lafayette | LA | 70508 |
| Ochsner Medical Center | 1514 Jefferson Hwy | New Orleans | LA | 70121 |
| LSU-ENT Cochlear Implant Team | 433 Bolivar Street New | Orleans | LA | 70112 |
| Ochsner LSU Health - Ambulatory Care Center | 1602 Kings Hwy | Shreveport | LA | 71103 |
| Beth Israel Deaconess Medical Ctr | 330 Brookline Ave | Boston | MA | 2215 |
| BMC Corporation | 830 Harrison Ave | Boston | MA | 2118 |
| Brigham and Women’s Hosp – Otolaryngology | 75 Francis St | Boston | MA | 2115 |
| Mass Eye & Ear – Main Office | 243 Charles St | Boston | MA | 2114 |
| Tufts Medical Center | 800 Washington St | Boston | MA | 2111 |
| VA Boston Healthcare System | 150 S Huntington Ave | Boston | MA | 2130 |
| Lahey Hospital & Medical Center | 41 Mall Rd | Burlington | MA | 1805 |
| Cambridge Health Alliance | 1493 Cambridge St | Cambridge | MA | 2139 |
| UMass Memorial Med Ctr | 55 Lake Ave N | Worcester | MA | 1655 |
| Baltimore VA Medical Center | 10 N Greene St | Baltimore | MD | 21201 |
| Greater Baltimore Medical Center | 6535 N Charles St | Baltimore | MD | 21204 |
| Johns Hopkins Outpatient Center | 601 N Caroline St | Baltimore | MD | 21287 |

| | | | | |
|---|---|---|---|---|
| University of Maryland Medical Center | 16 S Eutaw St | Baltimore | MD | 21201 |
| Johns Hopkins at Suburban Hospital | 6420 Rockledge Drive | Bethesda | MD | 20817 |
| Walter Reed Army Medical Center | 4954 North Palmer Rd | Bethesda | MD | 20889 |
| Centers for Advanced ENT Care – Gaithersburg | 818 W Diamond Ave | Gaithersburg | MD | 20878 |
| Kaiser Permanente Largo Medical Center | 1221 Mercantile Lane | Largo | MD | 20774 |
| Rockville Surgical Suites | 3200 Tower Oaks Blvd | Rockville | MD | 20852 |
| Advanced ENT Care – Silver Spring | 10801 Lockwood Drive | Silver Spring | MD | 20901 |
| Maine Medical Partners Otolaryngology | 92 Campus Dr | Scarborough | ME | 4074 |
| Univ of Michigan | 475 Market Place | Ann Arbor | MI | 48108 |
| Henry Ford Hospital | 2799 West Grand Blvd | Detroit | MI | 48202 |
| Michigan Ear Institute — Farmington Hills | 30055 Northwestern Hwy | Farmington Hills | MI | 48334 |
| Holland Hospital — Grand Haven | 15100 Whitaker Way | Grand Haven | MI | 49417 |
| Corewell Health Audiology | 100 Michigan St NE | Grand Rapids | MI | 49503 |
| Henry Ford — Pierson Clinic | 131 Kercheval Ave | Grosse Pointe Farms | MI | 48236 |
| Holland Hospital | 577 Michigan Ave | Holland | MI | 49423 |
| Michigan Ear Institute — Novi | 46325 West 12 Mile Road | Novi | MI | 48377 |
| Michigan Ear Institute — Royal Oak | 3555 W Thirteen Mile Rd | Royal Oak | MI | 48073 |
| Corewell Health Ear, Nose and Throat - St. Joseph | 2680 S Cleveland Ave | St Joseph | MI | 49085 |
| Allina Health – Apple Valley | 14655 Galaxie Ave | Apple Valley | MN | 55124 |
| Midwest ENT Specialists — Eagan | 3460 Promenade Ave | Eagan | MN | 55123 |
| M Health Fairview Clinics and Surgery Center - Maple Grove | 14500 99th Ave N. | Maple Grove | MN | 55369 |
| M Health Fairview Clinics and Specialty Center - Maplewood | 2945 Hazelwood St. | Maplewood | MN | 55109 |

| | | | | |
|---|---|---|---|---|
| M Health Fairview | 909 Fulton St SE | Minneapolis | MN | 55455 |
| VA Med Ctr – Minneapolis | One Veterans Dr | Minneapolis | MN | 55417 |
| M Health Fairview Clinic - Princeton | 919 Northland Dr | Princeton | MN | 55371 |
| Mayo Clinic – Rochester | 200 1st Street S.W. | Rochester | MN | 55905 |
| HealthPartners Como Clinic | 2500 Como Ave | Saint Paul | MN | 55108 |
| HealthPartners Specialty Center | 401 Phalen Blvd | Saint Paul | MN | 55130 |
| Allina Health – Bandana Square Clinic | 1021 Bandana Blvd | St. Paul | MN | 55108 |
| Midwest ENT Specialists — Vadnais Heights | 3590 Arcade Street | Vadnais Heights | MN | 55127 |
| Midwest ENT Specialists — Woodbury | 217 Radio Drive | Woodbury | MN | 55125 |
| Missouri Ear Nose & Throat Ctr | 1000 W. Nifong Blvd | Columbia | MO | 65203 |
| MU-ENT, Hearing and Balance Center | 525 N. Keene St. | Columbia | MO | 65201 |
| Ear Nose and Throat Center Ballas Rd | 450 N New Ballas | Creve Coeur | MO | 63141 |
| St. Louis VA Medical Ctr – John Cochran | 915 N Grand Blvd | Saint Louis | MO | 63106 |
| Washington University | 425 S Euclid Ave | Saint Louis | MO | 63110 |
| Christian Northeast Hospital | 11133 Dunn Rd | St. Louis | MO | 63136 |
| Jackson Ear Clinic | 290 E Layfair Dr | Flowood | MS | 39232 |
| Lakeland Medical - UMMC | 764 Lakeland Drive | Jackson | MS | 39216 |
| Billings Clinic | 2800 10th Ave N | Billings | MT | 59101 |
| Glacier Ear Nose and Throat | 160 Heritage Way | Kalispell | MT | 59901 |
| Rocky Mountain ENT (MT) | 700 West Kent | Missoula | MT | 59801 |
| UNC Hospitals Audiology at Cary | 115 Kildaire Park Drive | Cary | NC | 27518 |
| UNC Hospitals – Hearing & Voice Center | 2226 Nelson Highway | Chapel Hill | NC | 27517 |
| Charlotte ENT Associates (Fairview) | 6035 Fairview Road | Charlotte | NC | 28210 |

| | | | | |
|---|---|---|---|---|
| Charlotte ENT Associates (Concord) | 645 Amalia St NE | Concord | NC | 28025 |
| Duke Otolaryngology of South Durham | 234 Crooked Creek Parkway | Durham | NC | 27713 |
| Durham VA Medical Center | 508 Fulton St | Durham | NC | 27705 |
| Eastern Carolina ENT | 850 Johns Hopkins Drive | Greenville | NC | 27835 |
| Carolina Ear & Hearing Clinic | 5900 Six Forks Road | Raleigh | NC | 27609 |
| Duke Otolaryngology of Raleigh | 3480 Wake Forest Road | Raleigh | NC | 27609 |
| Wilmington ENT | 2311 Delaney Avenue | Wilmington | NC | 28403 |
| Wake Forest Univ Baptist Medical Center – Comp Rehab Plaza | 131 Miller Street | Winston-Salem | NC | 27157 |
| Sanford Clinic Fargo | 801 Broadway N | Fargo | ND | 58102 |
| Boys Town National Research Hospital | 14000 Boys Town Hospital Rd | Boys Town | NE | 68010 |
| Ear Specialists – Omaha | 9202 W Dodge Rd | Omaha | NE | 68114 |
| Dartmouth-Hitchcock Medical Ctr | 1 Medical Center Dr. | Lebanon | NH | 3756 |
| Cooper University Hospital | 1 Cooper Plaza | Camden | NJ | 8103 |
| Thomas Jefferson University – Cherry Hill | 2211 Chapel Avenue West | Cherry Hill | NJ | 8002 |
| Hackensack Meridian Health Hospitals | 30 Prospect Ave | Hackensack | NJ | 7601 |
| St. Barnabas Medical Center | 94 Old Short Hills Rd | Livingston | NJ | 7039 |
| Robert Wood Johnson Univ Hospital | 1 Robert Wood Johnson Pl | New Brunswick | NJ | 8901 |
| Overlook Hospital | 550 Central Ave | New Providence | NJ | 7974 |
| University Hospital of New Jersey | 140 Bergen St | Newark | NJ | 7103 |
| Univ of New Mexico Hospital | 2211 Lomas Blvd NE | Albuquerque | NM | 87102 |
| VA Med Ctr – Albuquerque | 1501 San Pedro Dr SE | Albuquerque | NM | 87108 |
| Sandoval Regional Medical Ctr | 3001 Broadmoor Blvd NE | Rio Rancho | NM | 87144 |

| | | | | |
|---|---|---|---|---|
| UNLV Health | 5320 S Rainbow Blvd | Las Vegas | NV | 89118 |
| Montefiore Medical Center (Moses Division OR) | Steuben Ave at Gun Hill Rd | Bronx | NY | 10467 |
| Bassett Healthcare | One Atwell Rd | Cooperstown | NY | 13326 |
| Westchester Med. Cochlear Implant Ctr | 19 Bradhurst Ave | Hawthorne | NY | 10532 |
| NYU Winthrop | 2nd St | Mineola | NY | 11501 |
| Columbia Univ Medical Center | 180 Ft Washington | New York | NY | 10032 |
| Lenox Hill Hospital – Midtown | 110 E 59th St | New York | NY | 10022 |
| Mount Sinai Hospital | 101 St | New York | NY | 10029 |
| New York Eye & Ear Infirmary | 310 E 22nd St | New York | NY | 10010 |
| New York Otolaryngology Group – Midtown | 36A E 36th St | New York | NY | 10016 |
| Northwell Health Manhattan Eye, Ear & Throat Hospital | 210 E 64th St | New York | NY | 10021 |
| NY Presbyterian Hospital | 622 W 168th St | New York | NY | 10032 |
| NYP David H. Koch Center | 432 E 69th St | New York | NY | 10065 |
| NYP Weill Cornell Medical College | 1305 York Ave | New York | NY | 10021 |
| NYU Cochlear Implant Center | 222 E 41st St, 8th Fl | New York | NY | 10017 |
| Univ of Vermont – CVPH | 75 Beekman St | Plattsburgh | NY | 12901 |
| Univ of Rochester / Strong Memorial | 575 Elmwood Ave | Rochester | NY | 14642 |
| SUNY at Stony Brook | 37 Research Way | Setauket- East Setauket | NY | 11733 |
| Northwell Health Phelps Hospital | 701 North Broadway | Sleepy Hollow | NY | 10591 |
| Westchester Medical Center | 100 Woods Rd | Valhalla | NY | 10595 |
| NYU Langone Otolaryngology Assoc. | 173 Froehlich Farm Blvd | Woodbury | NY | 11797 |
| The University Hospital | 234 Goodman Street | Cincinnati | OH | 45219 |
| Univ of Cincinnati Medical Center - ENT | 3113 Bellevue Ave | Cincinnati | OH | 45219 |

| | | | | |
|---|---|---|---|---|
| VA Medical Center Cincinnati | 4600 Beechwood Rd | Cincinnati | OH | 45244 |
| MetroHealth Medical Center | 2500 Metrohealth Drive | Cleveland | OH | 44109 |
| University Hospitals of Cleveland | 1100 Euclid Ave | Cleveland | OH | 44106 |
| Ohio State Medical Center | 410 West 10th Ave | Columbus | OH | 43210 |
| OSU Hearing Professionals Research Dept. | 915 Olentangy River Road | Columbus | OH | 43212 |
| ProMedica Flower Hospital | 300 N Summit Street | Toledo | OH | 43604 |
| Toledo Hospital | 2142 North Cove Blvd | Toledo | OH | 43606 |
| Oklahoma Otolaryngology Associates - Edmond | 3824 S. Boulevard | Edmond | OK | 73013 |
| Oklahoma Otolaryngology Associates - Norman | 3650 W Rock Creek Rd | Norman | OK | 73072 |
| Oklahoma Ear Institute | 825 NE 10th Street | Oklahoma City | OK | 73104 |
| Central Oregon ENT | 2450 NE Mary Rose Pl | Bend | OR | 97701 |
| Northwest Ear Institute | 2230 NW Pettygrove St | Portland | OR | 97210 |
| Oregon Health & Science University | 3181 SW Sam Jackson Park Rd | Portland | OR | 97201 |
| The Oregon Clinic | 9155 SW Barnes Rd | Portland | OR | 97225 |
| Lehigh Valley Health Network | 1245 S Cedar Crest Blvd | Allentown | PA | 18103 |
| Hershey Medical Center | 500 University Dr | Hershey | PA | 17033 |
| Hershey Med Ctr – Penn State Lime Springs | 2221 Noll Dr | Lancaster | PA | 17603 |
| Penn Medicine – Tuttleman Center | 1840 South Street | Philadelphia | PA | 19146 |
| Thomas Jefferson University | 1101 Chestnut St | Philadelphia | PA | 19107 |
| Univ of Pennsylvania Health System | 3400 Civic Center Blvd | Philadelphia | PA | 19104 |
| VA Medical Center Philadelphia | 3900 Woodland Ave | Philadelphia | PA | 19104 |
| Univ of Pittsburgh Med Ctr | 203 Lothrop St | Pittsburgh | PA | 15213 |
| UPMC Mercy Hospital | 1400 Locust Street, | Pittsburgh | PA | 15219 |

| | | | | |
|---|---|---|---|---|
| Univ of Pennsylvania Health System – Radnor | 145 King of Prussia Rd | Radnor | PA | 19087 |
| University Otolaryngology | 1351 South County Trail | East Greenwich | RI | 2818 |
| University Otolaryngology | 830 Eddy Street | Providence | RI | 2905 |
| MUSC – Cochlear Implant Program | 135 Rutledge Ave | Charleston | SC | 29425 |
| Ralph H. Johnson VA Medical Center | 109 Bee Street | Charleston | SC | 29401 |
| Univ of South Carolina – Montgomery Speech-Language-Hearing Center | 1705 College Street | Columbia | SC | 29208 |
| Greenville ENT | 124 Verdae Blvd | Greenville | SC | 29605 |
| Prisma Upstate ENT | 200 Patewood Dr | Greenville | SC | 29615 |
| West River ENT at the Medical Center | 241 Minnesota St | Rapid City | SD | 57701 |
| Avera Medical Group ENT — Head & Neck Surgery — Plaza 1 | 1417 S Cliff Ave | Sioux Falls | SD | 57105 |
| Midwest Ear Nose & Throat | 2315 W 57th St | Sioux Falls | SD | 57108 |
| Yankton Medical Clinic, P.C. | 1104 West Eighth Street | Yankton | SD | 57078 |
| Franklin Surgery Center | 9160 Carothers Pkwy | Franklin | TN | 37067 |
| ENT Consultants of East TN | 9430 Park West Blvd | Knoxville | TN | 37923 |
| Farragut ENT and Allergy | 144 Concord Rd | Knoxville | TN | 37934 |
| Parkwest Medical Center | 9352 Park West Blvd | Knoxville | TN | 37923 |
| Vanderbilt Wilson County Hospital | 1411 West Baddour Pkwy | Lebanon | TN | 37087 |
| VA Med Ctr Memphis | 1030 Jefferson Ave. | Memphis | TN | 38104 |
| Murfreesboro Medical Clinic | 1272 Garrison Dr. | Murfreesboro | TN | 37129 |
| Otolaryngology Associates of Tennessee | 410 42nd Ave N | Nashville | TN | 37209 |
| VA Med Ctr Nashville | 1310 24th Ave S | Nashville | TN | 37212 |
| Vanderbilt Univ Medical Center | 1215 21st Ave South | Nashville | TN | 37232 |
| Southeast Texas Ear, Nose & Throat | 740 Hospital Dr. | Beaumont | TX | 77701 |

| | | | | |
|---|---|---|---|---|
| Texas ENT and Allergy | 1730 Birmingham Dr. | College Station | TX | 77845 |
| Univ of North Texas, Dept of Speech & Hearing Sci. | 907 W Sycamore St | Denton | TX | 76201 |
| Dallas Ear Institute – Frisco | 4461 Coit Rd | Frisco | TX | 75035 |
| Univ of Texas Medical Branch | 301 University Blvd, | Galveston | TX | 77555 |
| Baylor College of Medicine | 1977 Butler Blvd. | Houston | TX | 77030 |
| UT Physicians | 6400 Fannin St | Houston | TX | 77030 |
| VA Med Ctr – Houston | 2002 Holcombe Blvd | Houston | TX | 77030 |
| Univ of Texas Medical Branch – League City | 1600 W. League City Pkwy | League City | TX | 77573 |
| Ear Institute of Texas | 18518 Hardy Oak Blvd | San Antonio | TX | 78258 |
| Ear Medical Group | 21 Spurs Lane | San Antonio | TX | 78240 |
| University of Utah Hospital | 50 N. Medical Drive | Salt Lake City | UT | 84132 |
| Lone Peak Surgery Center | 96 Kimballs Ln | Draper | UT | 84020 |
| West Jordan South Valley ENT | 3584 W 9000 S | Jordan | UT | 84088 |
| Peak ENT | 3668 W 2150 N | Lehi | UT | 84043 |
| Ogden Clinic Professional Corp | 4650 Harrison Blvd | Ogden | UT | 84403 |
| Intermountain Health Utah Valley Hospital | 1034 N 500 W | Provo | UT | 84604 |
| Intermountain Health Riverton Hospital | 3741 W 12600 S | Riverton | UT | 84065 |
| Intermountain Health LDS Hospital | 8th Ave | Salt Lake City | UT | 84143 |
| VA Med Ctr – Salt Lake City | 500 Foothill Blvd | Salt Lake City | UT | 84148 |
| Spanish Fork Hospital | 765 E Mkt Pl Dr | Spanish Fork | UT | 84660 |
| CommonSpirit Holy Cross Hospital - West Valley | 3460 S 4155 W | West Valley City | UT | 84120 |
| Naval Medical Center Portsmouth | 54 Lewis Minor St | Portsmouth | VA | 23708 |
| VCU Cochlear Implant Center | 401 N. 11th Street | Richmond | VA | 23298 |

| | | | | |
|---|---|---|---|---|
| Univ of Vermont Medical Center | 111 Colchester Ave | Burlington | VT | 5401 |
| Swedish Medical Center (Edmonds) | 21601 76th Ave W | Edmonds | WA | 98026 |
| Swedish Medical Center (Issaquah) | 751 NE Blakely Dr | Issaquah | WA | 98029 |
| Ear Nose Throat & Allergy Associates | 104 27th Ave SE | Puyallup | WA | 98374 |
| First Hill Surgery Center | 1101 Madison St | Seattle | WA | 98104 |
| Kaiser Washington Capitol Hill Main Bldg | 201 16th Ave E | Seattle | WA | 98112 |
| Proliance Center for Specialty Surgery - Puget Sound ENT | 515 Minor Ave | Seattle | WA | 98104 |
| Swedish Medical Center (Cherry Hill) | 747 Broadway | Seattle | WA | 98122 |
| Swedish Medical Center (First Hill) | 702 Minor Ave | Seattle | WA | 98104 |
| UW Medical Center | 1959 NE Pacific St | Seattle | WA | 98195 |
| VA Med Ctr Seattle (Puget Sound HCS) | 1660 S Columbian Way | Seattle | WA | 98108 |
| Virginia Mason Medical Center | 1201 Terry Ave | Seattle | WA | 98101 |
| Columbia Surgery Center, LLC | 217 W Cataldo Ave | Spokane | WA | 99201 |
| Sacred Heart Medical Center | 101 W 8th Ave | Spokane | WA | 99204 |
| Mayo Clinic – Eau Claire | 1221 Whipple St | Eau Claire | WI | 54701 |
| St. Vincent Hospital | 835 S Van Buren St | Green Bay | WI | 54301 |
| Aurora Health Center – 84 South | 9000 W Sura Ln | Greenfield | WI | 53228 |
| VA Med Ctr – Middleton Memorial (Madison) | 2500 Overlook Terrace | Madison | WI | 53705 |
| Marshfield Clinic Health System | 1000 N Oak Ave | Marshfield | WI | 54449 |
| Aurora St. Luke’s Medical Center | 2900 W Oklahoma Ave | Milwaukee | WI | 53215 |
| Froedtert & the Medical College of WI | 9200 W Wisconsin Ave | Milwaukee | WI | 53226 |
| Milwaukee VA Medical Center | 5000 W National Ave | Milwaukee | WI | 53295 |
| CAMC Pediatric and Adult ENT | 830 Pennsylvania Ave | Charleston | WV | 25302 |

| | | | | |
|---|---|---|---|---|
| Mountain Ears Hearing Clinic | 3901 Briscoe Rd | Parkersburg | WV | 26101 |
